# Copy Number Variation of Immune-Related Genes and Their Association with Iodine in Adults with Autoimmune Thyroid Diseases

**DOI:** 10.1155/2018/1705478

**Published:** 2018-03-11

**Authors:** Xing Jin, Yunfeng Guan, Hongmei Shen, Yi Pang, Lixiang Liu, Qingzhen Jia, Fangang Meng, Xiaoye Zhang

**Affiliations:** ^1^Center for Endemic Disease Control, Chinese Center for Disease Control and Prevention, Harbin Medical University, Harbin, Heilongjiang, China; ^2^Institute for Endemic Disease Prevention and Treatment of Shanxi Province, Linfen, Shanxi, China

## Abstract

**Background:**

Autoimmune thyroid diseases (AITD) are complex conditions that are caused by an interaction between genetic susceptibility and environmental triggers. Iodine is already known to be an environmental trigger for AITD, but genes associated with susceptibility need to be further assessed. Therefore, the aims of this study were to assess the association between copy number variations (CNVs) and AITD, to identify genes related with susceptibility to AITD, and to investigate the interaction between iodine status and CNVs in the occurrence of AITD.

**Methods:**

Blood samples from 15 patients with AITD and 15 controls were assessed by chromosome microarray to identify candidate genes. The copy number of candidate genes and urinary iodine level was determined in adults from areas of different iodine statuses including 158 patients and 181 controls.

**Results:**

The immune-related genes, *SIRPB1* and *TMEM91*, were selected as candidate genes. The distribution of *SIRPB1* CNV in AITD patients and controls was significantly different and was considered a risk factor for AITD. There was no significant association between urinary iodine level and candidate gene CNVs.

**Conclusion:**

*SIRPB1* CNV and an excess of iodine were risk factors for AITD, but an association with the occurrence of AITD was not found.

## 1. Introduction

Autoimmune thyroid disease (AITD) is an organ-specific autoimmune disease that is characterized by the variable degree of lymphocytic infiltration of the thyroid gland, which can eventually lead to thyroid atrophy and loss of function [1, 2]. AITD affects about 5% of the overall population, and its prevalence varies among different populations [3, 4]. This suggests that there are factors that influence the susceptibility of different populations to the development of AITD, which implies an important genetic contribution.

Copy number variation (CNV) is a genetic variant caused by the rearrangement of the genome [5]. CNVs are distributed widely in the human genome and can make genetic variations more diverse [6]. Theoretically, the impact of CNVs may be far more extensive than that of single-nucleotide polymorphisms (SNPs), since deleted or extra copies of important genes may cause under- or overproduction of the corresponding protein, affecting the finely balanced biochemistry of the cell [7]. Therefore, it is not surprising that copy number gains or losses are critical genetic factors for diseases. To date, there have been many studies demonstrating that CNVs play important roles in the pathogenetic mechanism of complex diseases, but fewer about their relation with AITD [8–10].

Iodine plays a key role in the synthesis of thyroid hormones. An appropriate iodine status is crucial in maintaining normal thyroid morphology and function [11]. However, constant iodine prophylaxis and environmental iodine exposure potentially increase the risk of iodine excess [1]. Multiple studies have confirmed that iodine excess is an important environmental etiology for the development of AITD [12].

As with most other autoimmune disorders, the susceptibility to AITD is determined by interactions between genetic and environmental factors [13, 14]. Thus, one aim of this research was to discover if there were associations between CNVs and AITD and to identify new genes that contribute to the susceptibility of AITD. Another aim was to explore whether iodine and the CNVs of genes involved in susceptibility have significant associations with the occurrence of AITD.

## 2. Materials and Methods

### 2.1. Subjects

The subjects were recruited from the Maxi, Xiwenzhuang and Gaoche Villages of Shanxi Province in China, where iodine availability was adequate and sufficient and in excess. In total, 901 adults from the three areas were included in the AITD screening. A final total of 158 AITD patients enrolled in this study, and 181 healthy controls without thyroid disease matched for gender and age were also included. Among these participants, 58 patients and 81 controls were from the iodine-adequate area, 60 patients and 60 controls were from the iodine-sufficient area, and 40 patients and 40 controls were from the area where iodine was in excess. All AITD patients were identified by testing the levels of thyroglobulin antibody (TgAb) and thyroid peroxidase antibody (TPOAb).

The study was approved by the Ethics Committee of the Harbin Medical University, Harbin City, Heilongjiang Province, People's Republic of China, and is congruent with the Declaration of Helsinki. Subjects that participated in this study all signed an informed consent.

### 2.2. Methods

#### 2.2.1. Demographic Characteristics

All subjects were asked to fill in a questionnaire to acquire demographic information including name, age, height, and weight.

#### 2.2.2. TgAb and TPOAb Detection

Nonanticoagulative blood samples were centrifuged, and serum samples were obtained for testing. TgAb and TPOAb levels were determined using a chemiluminescent immunoassay (Roche Diagnostics GmbH, Mannheim, Germany). The reference values were 0–34 IU/mL (TPOAb) and 0–115 IU/mL (TgAb). The diagnostic criteria for AITD were TPOAb > 34 IU/mL and/or TgAb > 115 IU/mL.

#### 2.2.3. Urinary Iodine Detection

Spot urine samples were collected from all of the subjects and stored in clean plastic tubes at 4°C. Urinary iodine concentrations were measured according to China health standard method for the determination of iodine in urine by As^3+^-Ce^4+^ catalytic spectrophotometry [15].

#### 2.2.4. DNA Preparation

Anticoagulative blood samples were stored at −80°C for DNA extraction. An E.Z.N.A.® Blood DNA Kit (Omega Bio-tek, Norcross, Georgia, USA) was used to extract genomic DNA as described by the manufacturer. The purity of DNA was verified by the ratio A260/A280 = 1.8–2.0.

#### 2.2.5. Chromosome Microarray

DNA extracted from the blood samples of five AITD patients and five controls from each area were assessed by chromosome microarray on the Affymetrix CytoScan™ HD platform (Santa Clara, CA, USA) according to the manufacturer's specifications. These arrays contain more than 2.6 million copy number markers of which 750,000 are SNPs and 1.9 million are nonpolymorphic probes. The manufacturer's protocol was followed for amplification, hybridization, washing, and staining steps. Copy number analysis was performed using Affymetrix Chromosome Analysis Suite (ChAS) software.

#### 2.2.6. Real-Time PCR

The copy numbers of candidate genes, signal regulatory protein beta 1 gene (SIRPB1) and transmembrane protein 91 gene (TMEM91), were quantified for each subject using the real-time PCR method. Real-time PCR was performed on the ABI 7500 Fast System (Applied Biosystems, Foster City, CA, USA) using Fast-Start Universal SYBR Green Master (Roche Diagnostics GmbH, Mannheim, Germany). Primers for the candidate genes were designed by online Primer 3.0 software based on the sequence data obtained from the NCBI database. The sequences of primers were as follows: *SIRPB1*, forward primer: 5′-GGAATTGAGAGGCTGCTGTG-3′ and reverse primer: 5′-TTCCCAAGACAGGCAGGATT-3′ and *TMEM91*, forward primer: 5′-TGCT GCCTTGGGATTCCTTA-3′ and reverse primer: 5′-AGCAGGCTCATGACTCACTT-3′. Each sample was run in triplicate, and copy number estimate was derived by the comparative Ct method (ΔΔCt) using a single-copy control gene *RPPH1* (forward primer: 5′-AGCTGAGTGCGTCCTGTCACT-3′, reverse primer: 5′-TCTGGCCCTA GTCTCAGACCTT-3′) and a reference genomic DNA of a known copy number. Copy numbers ≤ 1.5 were considered to indicate losses, and those ≥ 2.5 were considered gains [16].

#### 2.2.7. Statistical Analysis

Normally distributed data were expressed as the mean values and standard deviations, whereas skew-distributed data were expressed as the median of the 25th and 75th percentiles. In the comparison of the groups, variables distributed normally were assessed with a group *t*-test and variables with a skew distribution were assessed with a Kruskal–Wallis test. Group comparisons of categorical variables were performed by the chi-square test. Spearman's rho was used to evaluate the associations among gene CNVs. Multiple logistic regression analysis was applied to evaluate the risk of AITD and the associations between iodine and CNVs. All statistical analyses were performed using SPSS version 17.0 for Windows. A *p* value lower than 0.05 was considered statistically significant.

## 3. Results

### 3.1. Demographic Characteristics

The demographic characteristics of adults in the three areas are presented in Table 1. Median urinary iodine values in the iodine-sufficient and iodine-excess groups were significantly higher than those in the iodine-adequate group (218.60 and 418.65 versus 126.90 *μ*g/L). However, the others did not reach statistical significance among the three groups.

The demographic characteristics of AITD patients and controls are presented in Table 2. There were no statistical significances in terms of age, sex and body mass index between the case and the control groups. Compared with the control group, median urinary iodine value was significantly higher in the case group (225.40 versus 176.05 *μ*g/L).

### 3.2. Analysis of Chromosome Microarray

**Figure 1:**
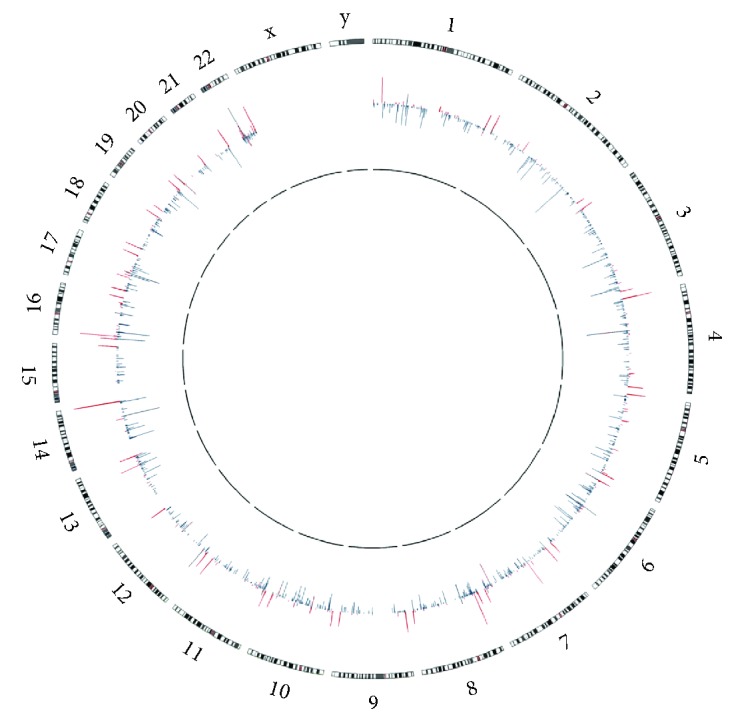
Genome-wide distribution of CNV genes. The outer circle represented chromosome numbers and ideograms; the inner circle represented copy number variations at corresponding chromosome loci. Outward red bars depicted amplifications, and inward green bars depicted deletions. The graph was plotted using Circos.

A total of 5616 CNV segments were identified in the 30 samples including 1925 gains and 3691 losses (Figure 1). There was a mean of 64 gains and 123 losses per genome. The size of CNV gains spanned from 8 bp to 4.8 Mb, while the size of CNV losses ranged from 8 bp to 3.4 Mb. According to a genome-wide association study based on the comparison of patients and controls, two immune-related genes, *SIRPB1* and *TMEM91*, were selected as candidate genes, which were located on the chromosomal regions 20p13 and 19q13.2, respectively. Obvious differences between the CNVs of the two genes were detected between patients and controls. The numbers of *SIRPB1* and *TMEM91* CNVs that occurred in the patients were nine and four, respectively; however, the numbers in the controls were five and zero.

### 3.3. The Distribution of Candidate Gene CNVs in the Population

The distributions of *SIRPB1* and *TMEM91* CNVs in AITD patients and controls are summarized in Table 3. There was an association between *SIRPB1* CNV and AITD; the frequency of CNV gain for *SIRPB1* gene in AITD patients was significantly higher than the controls (*χ*^2^ = 10.150, *p* = 0.001). However, there was no significant association between *TMEM91* CNV and AITD.

### 3.4. Relationship between Candidate Gene CNVs

The copy number of *SIRPB1* and *TMEM91* for 158 AITD patients was used to explore the relationship between the CNVs of the two genes. According to the analysis of Spearman's rho, a significant correlation was found between *SIRPB1* and *TMEM91* CNVs (coefficient correlation, *r*_s_ = 0.292, *p* < 0.001).

### 3.5. Analysis of Risk Factors for AITD in Adults

Analysis of multivariate logistic regression revealed that high urinary iodine levels (odds ratio (OR) = 1.94, *p* = 0.031) and *SIRPB1* CNVs (OR = 3.51, *p* = 0.016) were risk factors for AITD (Table 4).

### 3.6. Association between Candidate Gene CNVs and Urinary Iodine Level

As shown in Table 5, there were no significant associations between urinary iodine level and the CNVs of *SIRPB1* and *TMEM91*.

## 4. Discussion

CNV can influence the susceptibility to disease and explain genetic pathogeny as related studies have been widely applied in the etiology of complex chronic diseases [5]. Previous CNV studies are mainly related to cancer and chronic diseases [17–20]; however, AITD is seldom researched. In this study, blood samples from AITD patients and controls were analyzed by means of chromosome microarray, and CNVs were found to exist in numerous gene loci. This might suggest an association between AITD and CNVs. Thus, further research is needed to verify this. Increasing data have suggested that immune-related gene mutations play a key role in autoimmune diseases [14, 21]. Therefore, two immune-related genes were selected as candidate genes according to the results of the microarray analysis. To the best of our knowledge, this is the first study to show the effects of the CNV of these two genes on AITD.

The protein encoded by *SIRPB1* is a member of the signal regulatory protein family and also belongs to the immunoglobulin superfamily [22]. Previous studies on *SIRPB1* mainly focus on its biochemical characteristics and functions, finding that it can not only promote phagocytosis in macrophages but can also activate the mitogen-activated protein kinase (MAPK) pathway that regulates various functions [22, 23]. The MAPK pathway can be activated by multiple cytokines and participate in the occurrence and development of thyroid diseases [24–26]. In the present study, a statistically significant association between the CNV of the *SIRPB1* gene and AITD was identified. Genetic variation within the *SIRPB1* region may influence the secretion of inflammatory mediators and chemokines via the MAPK pathway, which could initiate or exacerbate the autoimmune response against the thyroid gland in AITD [27, 28]. However, this hypothesis has not been verified and requires further research.

*TMEM91* encodes a protein belonging to the transmembrane protein family which mediates a number of human physiological processes, such as the regulation of cell migration and invasion, constructs ion channels, and participates in the immune response [29–31]. At present, research on the TMEM family members focuses mostly on the field of cancer; however, their relationship with thyroid disease is unknown [32]. In this study, the association between *TMEM91* and AITD was investigated for the first time, but the results indicate that the association between the CNV of *TMEM91* and AITD was not statistically significant. Nevertheless, there existed a significant correlation between CNVs of *TMEM91* and *SIRPB1*, indicating that the small study sample size may be the reason for these nonsignificant findings.

With the implementation of universal salt iodization in China, the spectrum of thyroid disease is now undergoing fundamental change. The prevalence of AITD is a trend that is increasing year by year [33]. An excess of iodine is known to be an environmental risk factor for AITD [1], which is confirmed by the present study. Although the mechanisms of AITD are still unclear, it is a multifactorial disease with susceptibility determined by a combination of genetic and environmental factors [34]. Therefore, the interaction between candidate gene CNVs and urinary iodine level was further assessed. However, no significant interaction was found between them. One reason might be the restriction in the number of samples. Another possible reason is that the occurrence of CNVs increases the individuals' susceptibility to AITD; AITD may occur when susceptible individuals are exposed to environmental triggers such as infection, iodine, and stress [4, 13]. These factors working together could make the association between the CNV of genes and urinary iodine levels seem unremarkable. Hence, in future research, the mechanism of the interaction between genetic and environmental factors deserves further investigation, which will have a significant effect on the prevention of AITD.

The main strengths of this study are as follows. We explored the interaction between different iodine statuses and CNVs, which has rarely been reported. Furthermore, subjects with different iodine statuses enrolled in this study make the results more credible. However, there are some limitations in our study. Because the occurrence of AITD was limited in the samples of different iodine status areas, the copy number gains and losses were merged for analysis. Therefore, more cases need to be collected to further explore this. In addition, because of lacking clinical diagnosis, patients with Hashimoto thyroiditis or Graves' disease could not be easily distinguished; the patients might be a mixture of the two diseases. Hence, the relationship between CNVs and the phenotype of AITD should be further clarified in future studies. Lastly, with the restrictions of race, territory, and sample size in this research, the results do not represent general situations in China but will be the basis of a further study on genetic pathogeny.

## 5. Conclusion

The present study indicated that the CNV of the *SIRPB1* gene and an excess of iodine were risk factors for AITD, but an association with the occurrence of AITD was not discovered. Further research with a larger sample size is necessary to explore this.

## Figures and Tables

**Table 1:** Demographic characteristics of adults in three areas.

Characteristics	Maxi (iodine-adequate group)	Xiwenzhuang (iodine-sufficient group)	Gaoche (iodine-excess group)
*N*	324	286	291
Sex (*N*)			
Male	132	90	135
Female	192	196	156
Age			
Mean ± SD	46 ± 10	52 ± 13	45 ± 12
Range	19–70	18–80	20–66
BMI			
Mean ± SD	24.91 ± 3.51	25.30 ± 3.45	25.24 ± 3.61
UIC (*μ*g/L)			
P50	126.90	218.60^∗^	418.65^#^
P25-P75	87.70–209.08	146.80–302.60	277.90–605.85
TgAb-positive rate % (*n*/*N*)	13.58 (44/324)	15.73 (45/286)	9.97 (29/291)
TPOAb-positive rate % (*n*/*N*)	10.19 (33/324)	11.89 (34/286)	9.28 (27/291)
TgAb- and TPOAb-positive rate % (*n*/*N*)	5.86 (19/324)	6.64 (19/286)	5.50 (16/291)
AITD prevalence % (*n*/*N*)	17.90 (58/324)	20.98 (60/286)	13.75 (40/291)

BMI: body mass index; UIC: urinary iodine concentration. ^∗^Compared with the iodine-adequate group, median urinary iodine value was significantly higher in the iodine-sufficient group (*Z* = −8.291, *p* < 0.001). ^#^Compared with the iodine-adequate group, median urinary iodine value was significantly higher in the iodine-excess group (*Z* = −15.194, *p* < 0.001).

**Table 2:** Demographic characteristics of AITD patients and controls.

Characteristics	Case	Control
*N*	158	181
Sex (*N*)		
Male	35	35
Female	123	146
Age		
Mean ± SD	49 ± 12	47 ± 11
Range	18–76	20–77
BMI		
Mean ± SD	25.14 ± 3.45	24.57 ± 2.99
UIC (*μ*g/L)		
Median	225.40^∗^	176.05
Interquartile range	141.20–396.00	103.18–328.15

BMI: body mass index; UIC: urinary iodine concentration. ^∗^Compared with the control group, median urinary iodine value was significantly higher in the case group (*Z* = −2.554, *p* = 0.011).

**Table 3:** The distributions of *SIRPB1* and *TMEM91* CNVs in AITD patients and controls.

Gene/CNV	Case, *n* (%) *N* = 158	Control, *n* (%) *N* = 181	*p* value
SIRPB1			
Normal	138 (87.34)	174 (96.13)	
Loss	9 (5.70)	6 (3.32)	0.288
Gain	11 (6.96)	1 (0.55)	0.001^∗^
TMEM91			
Normal	148 (93.67)	176 (97.24)	
Loss	9 (5.70)	4 (2.21)	0.095
Gain	1 (0.63)	1 (0.55)	1.000

^∗^The frequency of CNV gain for *SIRPB1* gene in the case group was significantly higher than that in the control group.

**Table 4:** Multivariate logistic regression analysis of AITD on determinants^#^.

Variables	OR	95% CI	*p* value
UIC			
100 *μ*g/L < UIC ≤ 200 *μ*g/L	Ref		
UIC ≤ 100 *μ*g/L	0.86	0.42–1.75	0.678
200 *μ*g/L < UIC ≤ 300 *μ*g/L	1.64	0.84–3.21	0.149
UIC> 300 *μ*g/L	1.95	1.07–3.55	0.030^∗^
CNV			
SIRPB1	3.66	1.40–9.58	0.008^∗^
TMEM91	1.40	0.42–4.68	0.583

OR: odds ratio; CI: confidence interval. ^#^Adjusted for sex, age, and BMI. ^∗^Statistically significant results.

**Table 5:** The associations between candidate gene CNVs and urinary iodine level.

	100–200 *μ*g/L	<100 *μ*g/L	200–300 *μ*g/L	>300 *μ*g/L
*N*	92	62	68	103
Case	36	23	35	57
Control	56	39	33	46
SIRPB1				
OR^∗^	Ref	3.21	0.76	1.35
95% CI		0.21–49.38	0.04–13.67	0.15–12.42
TMEM91				
OR^∗^	Ref	2.08	1.12	2.30
95% CI		0.08–52.93	0.04–28.21	0.11–48.47

^∗^Adjusted for sex, age, and BMI.
